# Orthotics for First Metatarsophalangeal Osteoarthritis: Are we Asking the Wrong Question?

**DOI:** 10.1002/jfa2.70196

**Published:** 2026-08-02

**Authors:** Thomas W. Wainwright

**Affiliations:** ^1^ Orthopaedic Research Institute Bournemouth University Bournemouth UK; ^2^ University Hospitals Dorset NHS Foundation Trust Bournemouth UK

## Abstract

Orthotic interventions are commonly used in the management of first metatarsophalangeal (MTP) joint osteoarthritis, yet recent systematic review evidence suggests that their average treatment effects are small and unlikely to represent clinically meaningful benefit for the average patient. This has led to a growing perception that orthotics may have limited clinical value. However, such conclusions risk oversimplifying the evidence by conflating a lack of average treatment effect with a lack of utility for individual patients. This commentary explores this tension through critical appraisal of the current evidence base and consideration of treatment effect heterogeneity. It argues that responder heterogeneity, combined with the biomechanical specificity of first MTP osteoarthritis, may explain why orthotics provide substantial benefit for some patients despite weak average effects in trials. The discussion further highlights a mismatch between current osteoarthritis guidelines, which emphasise exercise as a core treatment, and the limited capacity for exercise to meaningfully influence joint loading in distal joints such as the first MTP. Drawing on evidence from hip osteoarthritis, including the CLEAT trial, the commentary contrasts capacity‐based and load‐modifying interventions and proposes a shift towards more mechanistically informed and individualised treatment strategies.

Recent systematic review evidence suggests that foot orthoses and shoe‐stiffening inserts provide only small, likely clinically unimportant benefits for pain and function in first metatarsophalangeal (MTP) joint osteoarthritis compared with sham interventions [1]. These findings are, of course, important and should inform clinical practice. However, there is a risk that “lack of a clinically meaningful average treatment effect” can be equated with “lack of clinical utility.”

This issue was recently brought into sharp focus through a personal experience of symptomatic improvement following the use of orthotic support during a flare of first MTP osteoarthritis. While anecdotal observations cannot be used to infer treatment effectiveness, they can prompt reflection on how substantial individual responses may coexist with small average treatment effects reported in clinical trials. This apparent discrepancy raises an important question: are we asking the right question when evaluating orthotic interventions?

The apparent contradiction is resolved when we consider the process of evaluating evidence across trials. The conclusion that “orthotics do not work” does not accurately reflect the underlying data. Rather, current evidence demonstrates that orthotics do not produce meaningful average benefits across heterogeneous patient populations. This distinction is critical, particularly in a condition as clinically variable as first MTP osteoarthritis, and where this variability is increasingly understood to reflect not only mechanical factors, but also the broader biological and metabolic processes that contribute to osteoarthritis symptom expression and progression.

First MTP osteoarthritis encompasses a spectrum of presentations, including differences in joint stiffness, dorsiflexion limitation, structural degeneration, and gait adaptations. From a mechanistic perspective, orthotic interventions may plausibly reduce joint loading, or dorsiflexion demands during gait, thereby modifying the mechanical environment of the joint and interacting with the underlying biological processes that contribute to symptom generation. It is therefore plausible that such interventions may be highly effective for a subset of patients while offering minimal benefit to others. When these subgroups are combined within randomised controlled trials, meaningful effects in responders may be diluted, resulting in small average treatment effects that mask clinically important individual responses.

This observation reflects a broader challenge within musculoskeletal research. Kerry has argued that clinical practice is characterised by complexity and context‐sensitive variation, whereas conventional research methods are largely designed to identify average effects across populations [2]. In this context, population‐level estimates of treatment effect may not fully capture the variability of individual responses observed in routine clinical practice. Rather than representing a contradiction, the coexistence of small average effects and substantial benefit in selected individuals may be an expected consequence of treatment effect heterogeneity within complex clinical systems.

This interpretation is consistent with the wider literature. Earlier systematic reviews have noted the scarcity of high‐quality trials in this area [3], while more recent narrative as well as observational work suggests that orthoses can provide symptom relief in some patients, albeit with methodological weaknesses and uncertainty regarding long‐term efficacy [4, 5]. Furthermore, the use of “sham” inserts as comparators may underestimate true treatment effects, as these interventions are unlikely to be biomechanically inert.

From a clinical perspective, this creates a tension between evidence‐based recommendations and patient‐centred care. Similar concerns have been raised within musculoskeletal research more broadly, where the translation of population‐level evidence into decisions for individual patients has been questioned [2]. The challenge is not that evidence from randomised trials lacks value, but that average treatment effects may not always provide sufficient information to support treatment decisions for individual patients whose clinical presentations differ substantially from those represented by the population mean. In practice, clinicians frequently observe variability in response to orthotic interventions. This variation is not inconsistent with the evidence but rather indicates its limitations. Evidence from randomised trials should inform, but not replace, clinical reasoning, particularly where interventions are low risk and easily reversible.

This tension is also reflected in contemporary clinical guidelines. The National Institute for Health and Care Excellence (NICE) [6] recommends therapeutic exercise for all people with osteoarthritis, irrespective of joint site, supported by strong evidence primarily derived from hip and knee populations. However, the applicability of this recommendation to distal joints such as the first MTP is less clear. Unlike larger joints, where improvements in muscle strength and neuromuscular control may meaningfully influence joint loading and symptom expression, the mechanisms through which exercise exerts benefit may not translate directly to the first MTP joint. In this setting, local joint loading is likely to be influenced more strongly by structural characteristics, footwear, and external load‐modifying interventions, potentially limiting the capacity of exercise alone to address joint‐specific mechanical drivers of symptoms. Therefore, while exercise remains important for general health and symptom modulation, exercise alone may be insufficient as a targeted intervention for joint‐specific symptom control in this context. This is particularly relevant given the growing recognition that osteoarthritis is not solely a mechanical condition, but one influenced by systemic and metabolic factors, which may not be directly addressed by joint‐specific exercise interventions alone.

This highlights a broader conceptual distinction between capacity‐based interventions (such as exercise and weight management) and load‐modifying interventions (such as orthoses and footwear adaptations). In larger joints, increasing physical capacity may improve load tolerance and reduce symptoms. For example, in hip osteoarthritis, interventions such as the CLEAT trial demonstrated improvements in pain and function through a group‐based cycling and education programme [7], potentially reflecting enhanced muscular support and improved movement patterns. However, even in this context, between‐group differences were modest and variability in response was evident. In contrast, for distal joints such as the first MTP, where loading is strongly influenced by joint structure and external interfaces, interventions that directly modify load may be more mechanistically aligned with symptom drivers. Within this contemporary model of osteoarthritis as a condition driven by both mechanical and biological factors, load‐modifying interventions may act not only by altering joint mechanics, but also by influencing downstream inflammatory and pain‐related pathways linked to joint stress.

This creates an important paradox within current evidence hierarchies. The intervention with the strongest evidence base (exercise) may be the least joint‐specific for first MTP osteoarthritis, while interventions that directly target joint loading (such as orthoses) have a weaker evidence base yet a plausible biomechanical rationale. Current trial designs, which prioritise average treatment effects across heterogeneous populations, may therefore underestimate the value of load‐modifying interventions in selected individuals.

A pragmatic “trial of treatment” approach may therefore be appropriate. Orthotics can be introduced with clear expectations, monitored over time, and continued or discontinued based on individual response. This is consistent with perspectives that position orthoses as a reasonable first‐line, low‐risk intervention within conservative management pathways [8]. Importantly, orthotic interventions are not only low risk, but also low cost, and place minimal demands on patient motivation, which may enhance adherence and real‐world effectiveness compared to interventions that require sustained behavioural change, such as structured exercise programmes. Orthotic interventions may also provide rapid symptom relief, allowing patients to maintain or resume walking with minimal delay. In this context, orthoses may function not only as load‐modifying devices, but as facilitators of physical activity, with potential downstream metabolic and systemic benefits.

For researchers, these findings highlight the need to move beyond the question of whether orthotics are effective, towards identifying which patients are most likely to benefit. Future studies should move beyond asking whether orthotics are effective on average and instead focus on identifying which patients are most likely to benefit. This will require greater emphasis on treatment‐effect heterogeneity, responder analyses, and the development and validation of robust clinical prediction and prognostic models capable of supporting personalised treatment decisions. Recent methodological guidance has highlighted both the opportunities and challenges of prediction modelling in musculoskeletal practice, emphasising that clinically useful models require rigorous development, validation and assessment of clinical utility before implementation [9, 10]. Greater use of objective outcome measures, including gait analysis and everyday activity monitoring, may also help to better characterise treatment response and identify clinically meaningful responder phenotypes.

More broadly, this discussion reflects a recurring challenge within musculoskeletal research. Average treatment effects derived from randomised trials may fail to fully represent the complexity of individual patient responses, particularly for interventions whose effects are likely to vary according to biomechanical, behavioural, and clinical characteristics. As others have argued, evidence generated from population‐based research must ultimately be interpreted within the context of individual patient care. The challenge for future research is therefore not simply determining whether orthotic interventions work on average but identifying responder phenotypes and clinical characteristics that predict meaningful benefit. Orthotics for first MTP osteoarthritis may not be universally effective. Perhaps the wrong question is whether orthotics work on average. The more clinically relevant question is which patients benefit, under what circumstances, and through which mechanisms.

## Author Contributions

**Thomas W. Wainwright:** conceptualization, writing – original draft, writing – review and editing.

## Funding

The author has nothing to report.

## Conflicts of Interest

The author declares no conflicts of interest.

## Data Availability

Data sharing not applicable to this article as no datasets were generated or analysed during the current study.
